# Predictive and Prognostic Factors of Synchronous Colorectal Lung-Limited Metastasis

**DOI:** 10.1155/2020/6131485

**Published:** 2020-11-23

**Authors:** Yuqiang Li, Zhongyi Zhou, Da Liu, Ming Zhou, Fengbo Tan, Wenxue Liu, Hong Zhu

**Affiliations:** ^1^Department of Gastrointestinal Surgery, Xiangya Hospital, Central South University, Changsha, China; ^2^Department of Thoracic Surgery 3 Zone, Cancer Center of Guangzhou Medical University, Guangzhou, China; ^3^Department of Rheumatology, Guangdong Provincial People's Hospital, Guangdong Academy of Medical Sciences, Guangzhou, China; ^4^Department of Cardiology, Xiangya Hospital, Central South University, Changsha, China; ^5^Department of Radiotherapy, Xiangya Hospital, Central South University, Changsha, China

## Abstract

**Aim:**

This study is aimed at investigating predictive and prognostic factors of synchronous colorectal lung-limited metastasis (SCLLM) based on The Surveillance, Epidemiology, and End Results (SEER) database.

**Methods:**

A multivariate logistic regression model was constructed to identify independent predictors of SCLLM. A multivariate Cox proportional hazards regression model was used to distinguish independent prognostic factors.

**Results:**

This study enrolled 168,007 colorectal cancer (CRC) patients without metastatic diseases and 1,298 cases with SCLLM. Eight features, involving race, tumor location, pathological grade, histological type, T stage, N stage, and tumor size as well as CEA, could be used as the independent predictors. As the nomogram shown, the T4 stage contributed the most to SCLLM, followed by the N2 stage, elevated CEA, and rectal cancer. A multivariate regression analysis discriminated 9 independent prognostic factors, including age, race, marital status, pathological grade, T stage, colectomy/proctectomy, chemotherapy, CEA, and TD. The prognostic nomogram illustrated that nonresection/NOS played as the poorest prognostic factor, followed by nonchemotherapy, ≥75-year old and T4 stage. The cumulative survival curves revealed the influence of each prognostic factor on survival after controlling the other variables.

**Conclusions:**

This study identified independent predictors and prognostic factors for SCLLM based on a large database of the United States. The predictors and prognostic factors can provide supporting evidence for the prevention and treatment of SCLLM.

## 1. Introduction

Colorectal cancer (CRC) ranks as the third most common malignancy in males and the second in females [1]. In spite of widespread early detection screening for CRC, approximate 25% of CRC patients are found to have distant metastases at the time of diagnosis [2, 3]. Moreover, metastasis is the main cause of high mortality among CRC patients [4].

Currently, there has been a continuous increase in the number of CRC patients diagnosed with pulmonary metastases, accounting for 32.9% of all metastatic CRCs (mCRCs) [5], after the widespread use of chest CT scans in recent years. Meanwhile, some research reported that 4-9% patients with CRC suffered from synchronous lung metastasis [6–8]. The retrospective data from China reported that lungs being the first metastatic site reached 24.5% among patients with mCRC [9]. Nevertheless, there is limited information to guide clinical practice in colorectal lung metastasis. It is a mainstream practice that the therapeutic strategy for colorectal liver metastases is applied to lung metastasis [10–12]. Undoubtedly, the treatment experience from colorectal liver metastasis is conducive to the rapid development of therapeutic strategy of colorectal lung metastasis. However, some scholars believe that there are differences involving the metastatic pattern between the colorectal liver and lung metastasis [13, 14]. Thus, it is important to further investigate the risk factors of colorectal lung metastasis. In addition, in order to exclude the interference from other metastatic sites, this study focused on synchronous colorectal lung-limited metastasis (SCLLM), which was defined as colorectal cancer with lung-limited metastases at the time of diagnosis.

SCLLM is considered less frequent due to the different metastatic route. The routine metastatic process of CRC involves discrete steps (CRC cancer cells initially migrate to the liver via the portal system, followed by the lungs and finally other locations) [15, 16], while the spread of metastatic CRC to the lungs, either in isolation or as the first of several distant sites, may be attributable to venous drainage which bypasses the portal system and instead enters systemic circulation [17]. Nevertheless, the frequency of synchronous lung metastasis increased significantly by a nearly 3-folds in the past decades [15].

Due to the rareness of SCLLM, a large public database is needed to explore this issue. The Surveillance, Epidemiology, and End Results (SEER) database is a kind of population-based cancer registration system of the USA taking 34.6% Americans into account, which can provide some necessary clinical data and be used to be an excellent database to explore issues regarding various cancers.

Therefore, this study is aimed at investigating predictive and prognostic factors of SCLLM based on SEER database.

## 2. Materials and Methods

### 2.1. Patients

This retrospective analysis used data from the SEER-linked database. The SEER program of the National Cancer Institute is an authoritative source of information on cancer incidence and survival in the United States (U.S.) with updated annually. SEER currently collects and publishes cancer incidence and survival data from population-based cancer registries covering approximately 34.6% of the U.S. population [18]. Data from SEER was used to identify patients with CRC diagnosed between 2010 and 2016, and 230,301 patients were diagnosed with colorectal adenocarcinoma (ICD-O-3: 8140, 8141, 8143, 8144, 8145, 8147, 8201, 8210, 8211, 8213, 8220, 8221, 8230, 8253, 8255, 8260, 8261, 8262, 8263, 8280, 8440, 8441, 8460, 8470, 9471, 8481, and 8490) between these years in total. Exclusion criteria: (1) without positive histology (*n* = 1,591); (2) autopsy/death certificate only cases and survival months = 0 (*n* = 12,460); (3) M1b, M1NOS, and metastases to other organs (*n* = 36,818); (4) incomplete information regarding stage T and stage N (*n* = 10,127). The final study sample contained 169,305 CRC patients, including 1,298 SCLLM patients.

For each patient, the following data was acquired: age at diagnosis, married status, insurance, gender, race, grade, histological type, T stage, N stage, regional nodes examined (RNE), CEA, surgery for primary tumor, surgery for hepatic metastasis, tumor deposits (TD), perineural invasion (PNI), radiotherapy, and chemotherapy. We defined colectomy/proctectomy with RNE ≥ 12 as standard colectomy/proctectomy and colectomy/proctectomy with RNE < 12/NOS as simplified colectomy/proctectomy.

### 2.2. Statistical Analysis

Intergroup comparisons were analyzed using Pearson's chi-square test and Mann-Whitney *U* test depending on the nature of the data. A multivariate logistic regression model was constructed, including all independent variables that showed statistical significance on univariate analysis, to identify independent predictors of SCLLM. Meanwhile, a multivariate Cox proportional hazards regression model was used to distinguish independent prognostic factors. Univariate analysis of variables with significant differences was included in the Cox regression model for multivariate analysis. Cumulative survival function was also calculated by the multivariate Cox analysis for comparing the effect of each independent prognostic factor. Statistical analyses were performed using IBM SPSS statistics trial ver. 25.0 (IBM, Armonk, NY, USA). All reported *p* values lower than 0.05 were considered significant.

## 3. Results

### 3.1. Patient Characteristics

This study enrolled 168,007 CRC patients without metastatic diseases and 1,298 cases with SCLLM. The entire cohort was predominantly elderly (≥65, 58.07%) and white people (75.27%). The rectum was the main site occurring lung-limited metastases in CRC. Besides, SCLLM was related to marital status, race, pathological grade, and histological type. Meanwhile, there were significant differences regarding the depth of tumor invasion and regional lymph node status between the two cohorts. Moreover, a lower rate of surgery but a significantly higher rate of chemotherapy and radiotherapy can be observed in the patients with SCLLM. Furthermore, SCLLM patients suffered a larger tumor size and a higher positive ratio of CEA, TD, and PNI, as well as a shorter median survival (Table 1).

### 3.2. Predictive Factors of Synchronous Colorectal Lung-Limited Metastasis

**Figure 1:**
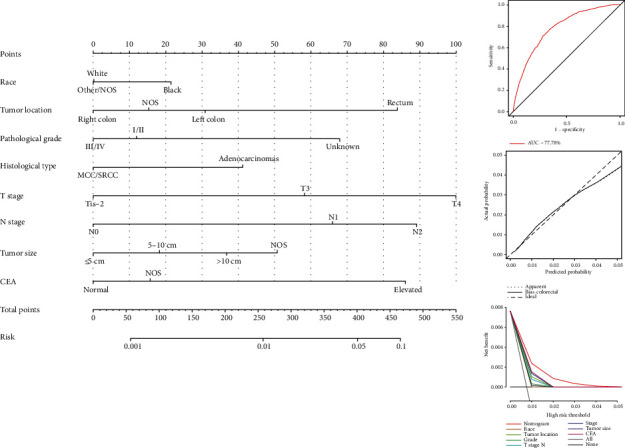
The weight of each independent predictor of SCLLM.

This section of the study excluded therapeutic variables and postoperative variables, including colectomy, pulmonary surgery, radiotherapy, chemotherapy, TD, and PNI. All variables with *p* values less than 0.05 in the univariate logistic regression model were brought into the multivariate regression analysis, which displayed that 8 features, involving race, tumor location, pathological grade, histological type, T stage, N stage, and tumor size as well as CEA, could be used as the independent predictors (Table 2). Furthermore, a nomogram was constructed to clearly show the weight of each independent predictor. As the nomogram shown, the T4 stage contributed the most to SCLLM, followed by the N2 stage, elevated CEA, and rectal cancer (Figure 1). Various methods, including ROC curves, calibration curves and decision curve analysis (DCA), were utilized to evaluate the discriminating superiority of the nomogram. The area under the curve (AUC) values of ROC were 77.78%. The calibration curves illustrated agreement between model prediction and actual observations. The DCA demonstrated net benefits of the nomogram and each prognostic factor.

### 3.3. Prognostic Factors of Synchronous Colorectal Lung-Limited Metastasis

**Figure 2:**
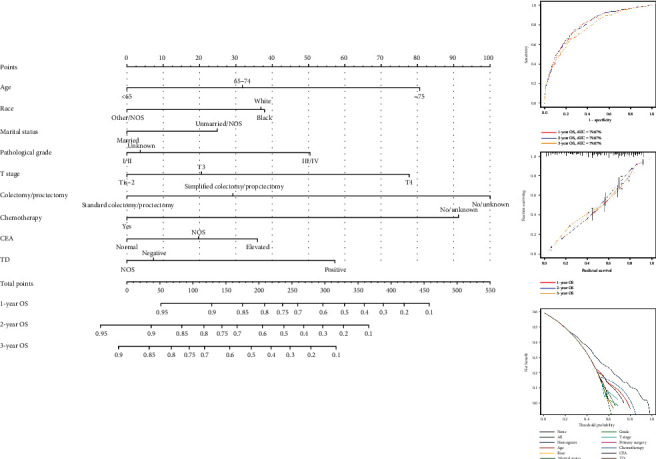
The impact of each prognostic factor on survival for patients with SCLLM.

**Figure 3:**
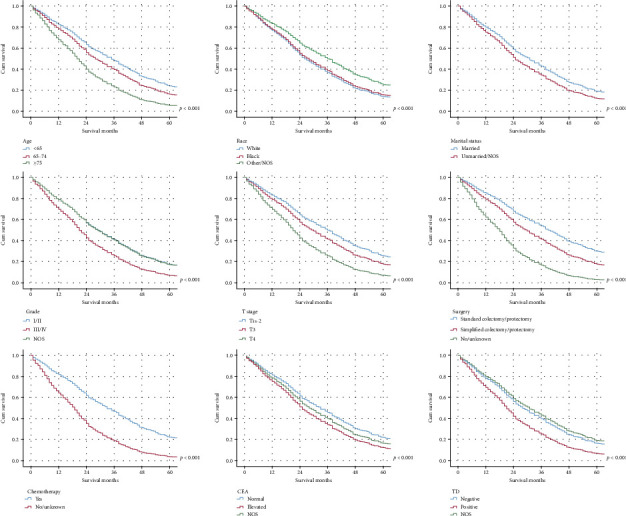
The cumulative survival curves revealed the influence of each prognostic factor on survival after controlling the other variables.

The qualified variables, that identified by a univariate Cox regression model, were further analyzed by a multivariate regression analysis, which discriminated 9 independent prognostic factors, including age, race, marital status, pathological grade, T stage, colectomy/proctectomy, chemotherapy, CEA, and TD (Table 3). In order to visually demonstrate the impact of each prognostic factor on survival, the cumulative survival curves and nomogram were utilized in accordance with the result of the multivariate Cox regression model. The prognostic nomogram illustrated that nonresection/NOS played as the poorest prognostic factor, followed by nonchemotherapy, ≥75-year-old and T4 stage (Figure 2). Meanwhile, the AUC values of ROC were 79.67%, 79.67%, and 76.97% regarding nomograms predicting 1-, 2-, and 3-year OS. The calibration curves demonstrated optimal agreement between model prediction and actual observations for 1-, 2-, and 3-year OS. The DCA indicated net benefits of the nomogram and each prognostic factor. Moreover, the cumulative survival curves revealed the influence of each prognostic factor on survival after controlling the other variables (Figure 3).

## 4. Discussion

To the best of our knowledge, this analysis was the first to look into the predictive and prognostic factors regarding OS for CRC with synchronous lung-limited metastasis. Colorectal oncologists have mainly focused on CRC with liver metastasis. Nevertheless, there is limited research on CRC with lung metastasis. The treatment of SCLLM commonly learns from the clinical experiences and strategies of treatment of colorectal hepatic metastasis [19]. In order to further improve treatment, it is essential to identify the specialized predictive and prognostic factors of SCLLM. CRC patients with high risk factors of lung metastasis should receive the particular treatments against prognostic factors and increase the frequency of follow-up.

Previous studies reported that the pattern of colorectal lung metastasis was the direct invasion of cancer cells into the systemic circulation through the veins [13], which was different from the method of colorectal liver metastasis, that was thought to result from the lymphatic drainage of the colon and rectum [14]. It may be the reason why the T stage can be used as both predictor and prognostic factor but the N stage can only play as a predictor of SCLLM. Moreover, numerous researches reported that TD was associated with reductions in survival [20, 21]. In fact, most of TD were thought to arise from lymphovascular invasion [22] and significantly related to T staging [22, 23]. Therefore, TD may be a manifestation of the ability and depth of tumor invasion affecting the survival of SCLLM patients.

RNE were considered as the priority for the assessment of the quality of surgery, which was mentioned in previous study [24], especially for the lack of the data concerning total mesorectal excision (TME) and complete mesocolic excision (CME) in the SEER database. The prognostic nomogram and survival curve manifested that standard colectomy/proctectomy with RNE ≥ 12 owned the clearest survival benefit comparing with noncolectomy and simplified resection. It is a consensus that high-quality colectomy/proctectomy means sufficient circumferential resection margin (CRM), which can be used as a specific therapeutic indicator against the depth of tumor invasion. Considering the critical role of T staging in patients with SCLLM, eligible TME/CME may be the most effective way to treat and prevent colorectal lung metastasis.

It is feasible to remove the primary tumor and liver metastasis in a simultaneous or staged approach for patients present with synchronous colorectal liver metastasis [25, 26]. Although existing some controversy concerning the order of resection of the liver metastasis and the primary tumor [19], none of synchronous, sequential liver-first, or bowel-first surgery appeared inferior to the others [25, 26]. Can the experience from colorectal liver metastasis be completely applied to SCLLM? The result of this study confirmed that independent pulmonary surgery, as a nonindependent prognostic factor in Cox regression analysis, did not improve the survival for SCLLM patients. Therefore, we believe that the approach of lung resection before resection of the primary tumor may be unreasonable for patients with SCLLM. Besides, more studies are needed to confirm whether the pulmonary surgery following by the colectomy/proctectomy cutting off the source of cancer cells and chemotherapy eliminating micrometastases can provide a survival benefit. In addition, CRC patients with metastatic diseases should receive radiation therapy cautiously [19]. This study believed that radiotherapy cannot improve survival for SCLLM patients as a whole. Nevertheless, it is meaningful to identify CRC patients who are sensitive to radiotherapy, as some other studies did [27, 28].

A growing body of data has shown that the location of the primary tumor can be both prognostic and predictive of response to EGFR inhibitors in metastatic colorectal cancer [29–31]. This study demonstrated inconsistent risk of lung-limited metastasis among right colon, left colon, and rectum. Several studies also proposed that rectal cancer is prone to metastasize to the lungs [15, 32]. Interestingly, there was no correlation between the primary site and the prognosis of patients with SCLLM. The mainstream opinions presently considered that targeted chemotherapy drugs, like cetuximab and panitumumab, improve survival for left-side colon patients but confer little benefit to right-side colon patients with metastatic diseases [29–31]. Does the consistent prognostic coefficient mean that the existing targeted drugs may not significantly prolong survival in all patients with SCLLM, including left colon and rectal cancer? It is uncertain and requires prospective research to verify.

A recent study involved the prognostic factors regarding cancer-specific survival for CRC with synchronous lung-limited metastasis [33]. However, study only focusing on cancer-specific survival inevitably misses some cases, such as those being not first tumor. Meanwhile, it is more reasonable to choose OS as the research endpoint since SCLLM, as a systemic disease, is able to affect the whole-body function. Limitations of this study include the following: (1) the use of retrospective data; (2) detailed treatment information for included patients were not recorded in the SEER cohort, and we could not investigate specific options, including chemotherapy regimen and specific surgical method, in the survival of SCLLM patients; and (3) the lack of some important genetic indicators, such as KRAS, NRAS, and BRAF. Future studies can focus on the molecular mechanisms of CRC with lung-limited metastasis.

## 5. Conclusion

This study identified independent predictors and prognostic factors for SCLLM based on a large database of the United States. The predictors and prognostic factors can provide supporting evidence for the prevention and treatment of SCLLM.

## Figures and Tables

**Table 1:** The characteristics of CRC patients associated with lung-limited metastasis.

Characteristics	Total (*n* = 169305)		Without lung-limited metastasis (*n* = 168007)		With lung-limited metastasis (*n* = 1298)		*p* value
	*n*	%	*n*	%	*n*	%	
Gender							0.899
Female	80313	47.44%	79695	47.44%	618	47.61%	
Male	88992	52.56%	88312	52.56%	680	52.39%	
Age (years)							0.072
<65	70997	41.93%	70425	41.92%	572	44.07%	
65-74	44114	26.06%	43776	26.06%	338	26.04%	
≥75	54194	32.01%	53806	32.03%	388	29.89%	
Marital status							0.001
Married	89491	52.86%	88863	52.89%	628	48.38%	
Unmarried/NOS	79814	47.14%	79144	47.11%	670	51.62%	
Insurance							0.141
Yes	160889	95.03%	159667	95.04%	1222	94.14%	
No/unknown	8416	4.97%	8340	4.96%	76	5.86%	
Race							0.010
White	133791	79.02%	132814	79.05%	977	75.27%	
Black	18894	11.16%	18711	11.14%	183	14.10%	
Other/NOS	16620	9.82%	16482	9.81%	138	10.63%	
Tumor location							<0.001
Right colon	72060	42.56%	71738	42.70%	322	24.81%	
Left colon	45969	27.15%	45677	27.19%	292	22.50%	
Rectum	49013	28.95%	48345	28.78%	668	51.46%	
NOS	2263	1.34%	2247	1.34%	16	1.23%	
Pathological grade							<0.001
I/II	130151	76.87%	129242	76.93%	909	70.03%	
III/IV	25628	15.14%	25427	15.13%	201	15.49%	
Unknown	13526	7.99%	13338	7.94%	188	14.48%	
Histological type							0.016
Adenocarcinomas	156108	92.21%	154888	92.19%	1220	93.99%	
MCC/SRCC	13197	7.79%	13119	7.81%	78	6.01%	
T stage							<0.001
Tis-2	65332	38.59%	65117	38.76%	215	16.56%	
T3	83185	49.13%	82444	49.07%	741	57.09%	
T4	20788	12.28%	20446	12.17%	342	26.35%	
N stage							<0.001
N0	110089	65.02%	109619	65.25%	470	36.21%	
N1	40665	24.02%	40144	23.89%	521	40.14%	
N2	18551	10.96%	18244	10.86%	307	23.65%	
Colectomy/proctectomy							<0.001
Standard resection	121185	71.58%	120545	71.75%	640	49.31%	
Simplified resection	26208	15.48%	26017	15.49%	191	14.71%	
Nonresection/NOS	21912	12.94%	21445	12.76%	467	35.98%	
Pulmonary surgery							<0.001
Yes	100	0.06%	0	0.00%	100	7.70%	
No/unknown	169205	99.94%	168007	100.00%	1198	92.30%	
Radiotherapy							<0.001
Yes	25351	14.97%	24993	14.88%	358	27.58%	
No/unknown	143954	85.03%	143014	85.12%	940	72.42%	
Chemotherapy							<0.001
Yes	59540	35.17%	58610	34.89%	930	71.65%	
No/unknown	109765	64.83%	109397	65.11%	368	28.35%	
Tumor size							<0.001
≤5 cm	101949	60.22%	101357	60.33%	592	45.61%	
5-10 cm	41599	24.57%	41177	24.51%	422	32.51%	
>10 cm	4149	2.45%	4092	2.44%	57	4.39%	
NOS	21608	12.76%	21381	12.73%	227	17.49%	
CEA							<0.001
Normal	59541	35.17%	59262	35.27%	279	21.49%	
Elevated	35452	20.94%	34835	20.73%	617	47.53%	
NOS	74312	43.89%	73910	43.99%	402	30.97%	
TD							<0.001
Negative	133508	78.86%	132910	79.11%	598	46.07%	
Positive	13672	8.08%	13448	8.00%	224	17.26%	
NOS	22125	13.07%	21649	12.89%	476	36.67%	
PNI							<0.001
Negative	132991	78.55%	132292	78.74%	699	53.85%	
Positive	13079	7.73%	12863	7.66%	216	16.64%	
NOS	23235	13.72%	22852	13.60%	383	29.51%	
Median survival (months)	30 (13-53)		30 (13-53)		18 (8-33)		<0.001

MCC: mucinous cell carcinoma; SRCC: signet ring cell carcinoma; CEA: carcinoembryonic antigen; TD: tumor deposits; PNI: perineural invasion; NOS: not otherwise specified.

**Table 2:** Univariable and multivariable logistic regression model analyses.

Characteristics	Univariable analysis				Multivariable analysis			
	OR	95% CI lower	95% CI upper	*p* value	OR	95% CI lower	95% CI upper	*p* value
Gender				0.899				
Female		Reference				NA		
Male	0.993	0.890	1.108	0.899				
Age (years)				0.197				
<65		Reference				NA		
65-74	0.951	0.831	1.088	0.462				
≥75	0.888	0.780	1.010	0.072				
Marital status				0.001				
Married		Reference				Reference		
Unmarried/NOS	1.198	1.074	1.336	0.001	1.112	0.995	1.243	0.062
Insurance				0.142				
Yes		Reference				NA		
No/unknown	1.191	0.943	1.503	0.142				
Race				0.001				0.021
White		Reference				Reference		
Black	1.330	1.135	1.558	<0.001	1.256	1.068	1.476	0.006
Other/NOS	1.138	0.952	1.361	0.156	1.004	.838	1.203	0.968
Tumor location				<0.001				<0.001
Right colon		Reference				Reference		
Left colon	1.424	1.215	1.669	<0.001	1.430	1.217	1.680	<0.001
Rectum	3.078	2.694	3.518	<0.001	2.633	2.287	3.031	<0.001
NOS	1.586	0.959	2.625	0.073	1.193	0.719	1.980	0.495
Pathological grade				<0.001				<0.001
I/II		Reference				Reference		
III/IV	1.124	0.964	1.310	0.135	0.871	0.743	1.023	0.092
Unknown	2.004	1.711	2.347	<0.001	1.900	1.603	2.251	<0.001
Histological type				0.016				<0.001
Adenocarcinomas		Reference				Reference		
MCC/SRCC	0.755	0.600	0.950	0.016	0.623	0.492	0.787	<0.001
T stage				<0.001				<0.001
Tis-2		Reference				Reference		
T3	2.722	2.338	3.170	<0.001	1.953	1.644	2.319	<0.001
T4	5.066	4.269	6.013	<0.001	3.143	2.579	3.831	<0.001
N stage				<0.001				<0.001
N0		Reference				Reference		
N1	3.027	2.671	3.431	<0.001	2.142	1.873	2.450	<0.001
N2	3.925	3.396	4.536	<0.001	2.797	2.388	3.277	<0.001
Tumor size				<0.001				<0.001
≤5 cm		Reference				Reference		
5-10 cm	1.755	1.548	1.989	<0.001	1.229	1.079	1.400	0.002
>10 cm	2.385	1.814	3.135	<0.001	1.518	1.144	2.015	0.004
NOS	1.818	1.559	2.120	<0.001	1.784	1.511	2.107	<0.001
CEA				<0.001				<0.001
Normal		Reference				Reference		
Elevated	3.762	3.264	4.336	<0.001	2.679	2.317	3.098	<0.001
NOS	1.155	0.991	1.346	0.065	1.194	1.023	1.394	0.025

MCC: mucinous cell carcinoma; SRCC: signet ring cell carcinoma; CEA: carcinoembryonic antigen; NOS: not otherwise specified; NA: unavailable.

**Table 3:** Univariable and multivariable Cox regression model.

Characteristics	Univariable analysis				Multivariable analysis			
	OR	95% CI lower	95% CI upper	*p* value	OR	95% CI lower	95% CI upper	*p* value
Gender				0.609				
Female		Reference				NA		
Male	1.039	0.898	1.203	0.609				
Age (years)				<0.001				<0.001
<65		Reference				Reference		
65-74	1.318	1.089	1.594	0.004	1.278	1.050	1.557	0.014
≥75	2.531	2.136	3.000	<0.001	2.014	1.663	2.440	<0.001
Marital status				<0.001				0.003
Married		Reference				Reference		
Unmarried/NOS	1.427	1.231	1.654	<0.001	1.263	1.082	1.475	0.003
Insurance								
Yes		Reference				NA		
No/unknown	1.126	0.830	1.527	0.447				
Race				0.040				0.035
White		Reference				Reference		
Black	0.866	0.700	1.071	0.185	0.950	0.760	1.188	0.653
Other/NOS	0.730	0.558	0.954	0.021	0.695	0.528	0.916	0.010
Tumor location				0.008				0.465
Right colon		Reference				Reference		
Left colon	0.742	0.600	0.916	0.006	0.930	0.746	1.158	0.515
Rectum	0.788	0.663	0.936	0.007	0.840	0.677	1.043	0.114
NOS	1.246	0.696	2.232	0.459	0.988	0.538	1.812	0.968
Pathological grade				<0.001				<0.001
I/II		Reference				Reference		
III/IV	1.426	1.172	1.734	<0.001	1.526	1.241	1.878	<0.001
Unknown	1.475	1.204	1.807	<0.001	1.011	0.808	1.266	0.920
Histological type				0.214				
Adenocarcinomas		Reference				NA		
MCC/SRCC	1.204	0.898	1.614	0.214				
T stage				<0.001				<0.001
Tis-2		Reference				Reference		
T3	0.746	0.612	0.909	0.004	1.268	1.000	1.607	0.050
T4	1.172	0.943	1.456	0.154	1.962	1.511	2.548	<0.001
N stage				0.036				0.169
N0		Reference				Reference		
N1	0.804	0.681	0.949	0.010	0.958	0.796	1.154	0.653
N2	0.901	0.743	1.092	0.287	1.168	0.925	1.476	0.193
Colectomy/proctectomy				<0.001				<0.001
Standard resection		Reference				Reference		
Simplified resection	1.294	1.041	1.608	0.020	1.434	1.138	1.805	0.002
Nonresection/NOS	1.914	1.631	2.246	<0.001	2.895	2.078	4.034	<0.001
Pulmonary surgery				<0.001				0.246
Yes		Reference				Reference		
No/unknown	2.061	1.512	2.808	<0.001	1.208	0.878	1.663	0.246
Radiotherapy				0.003				0.124
Yes		Reference				Reference		
No/unknown	1.289	1.090	1.523	0.003	1.172	.957	1.436	0.124
Chemotherapy				<0.001				<0.001
Yes		Reference				Reference		
No/unknown	2.694	2.314	3.137	<0.001	2.179	1.830	2.594	<0.001
Tumor size				<0.001				0.220
≤5 cm		Reference				Reference		
5-10 cm	1.144	0.966	1.355	0.119	1.069	0.898	1.272	0.454
>10 cm	2.040	1.466	2.838	<0.001	1.436	1.016	2.030	0.040
NOS	1.453	1.186	1.780	<0.001	1.104	0.877	1.390	0.401
CEA				0.004				0.006
Normal		Reference				Reference		
Elevated	1.376	1.129	1.675	0.002	1.381	1.128	1.692	0.002
NOS	1.362	1.106	1.676	0.004	1.182	.952	1.468	0.131
TD				<0.001				0.001
Negative		Reference				Reference		
Positive	1.493	1.216	1.832	<0.001	1.494	1.194	1.868	<0.001
NOS	1.807	1.535	2.128	<0.001	.908	.673	1.224	0.525
PNI				<0.001				0.404
Negative		Reference				Reference		
Positive	1.188	0.967	1.459	0.101	1.162	0.923	1.462	0.201
NOS	1.524	1.291	1.798	<0.001	1.060	0.867	1.297	0.569

MCC: mucinous cell carcinoma; SRCC: signet ring cell carcinoma; CEA: carcinoembryonic antigen; TD: tumor deposits; PNI: perineural invasion; NOS: not otherwise specified; NA: unavailable.

## Data Availability

These data were derived from the Surveillance, Epidemiology, and End Results (SEER) database (https://seer.cancer.gov/) and identified using the SEER^∗^Stat software (Version 8.3.5) (https://seer.cancer.gov/seerstat/).

## References

[1] Bray F., Ferlay J., Soerjomataram I., Siegel R. L., Torre L. A., Jemal A. (2018). Global cancer statistics 2018: GLOBOCAN estimates of incidence and mortality worldwide for 36 cancers in 185 countries. *CA: a Cancer Journal for Clinicians*.

[2] Amin M. B., Greene F. L., Edge S. B. (2017). The eighth edition AJCC cancer staging manual: continuing to build a bridge from a population-based to a more “personalized” approach to cancer staging. *CA: a Cancer Journal for Clinicians*.

[3] Kattan M. W., Hess K. R., Amin M. B. (2016). American Joint Committee on Cancer acceptance criteria for inclusion of risk models for individualized prognosis in the practice of precision medicine. *CA: a Cancer Journal for Clinicians*.

[4] Roth E. S., Fetzer D. T., Barron B. J., Joseph U. A., Gayed I. W., Wan D. Q. (2009). Does colon cancer ever metastasize to bone first? A temporal analysis of colorectal cancer progression. *BMC Cancer*.

[5] Wang Z., Wang X., Yuan J. (2018). Survival benefit of palliative local treatments and efficacy of different pharmacotherapies in colorectal cancer with lung metastasis: results from a large retrospective study. *Clinical Colorectal Cancer*.

[6] Farquharson A. L., Pranesh N., Witham G. (2008). A phase II study evaluating the use of concurrent mitomycin C and capecitabine in patients with advanced unresectable pseudomyxoma peritonei. *British Journal of Cancer*.

[7] Lieu C. H., Lambert L. A., Wolff R. A. (2012). Systemic chemotherapy and surgical cytoreduction for poorly differentiated and signet ring cell adenocarcinomas of the appendix. *Annals of Oncology*.

[8] Shapiro J. F., Chase J. L., Wolff R. A. (2010). Modern systemic chemotherapy in surgically unresectable neoplasms of appendiceal origin: a single-institution experience. *Cancer*.

[9] Li J., Yuan Y., Yang F. (2019). Expert consensus on multidisciplinary therapy of colorectal cancer with lung metastases (2019 edition). *Journal of Hematology & Oncology*.

[10] Tejani M. A., ter Veer A., Milne D. (2014). Systemic therapy for advanced appendiceal adenocarcinoma: an analysis from the NCCN Oncology Outcomes Database for colorectal cancer. *Journal of the National Comprehensive Cancer Network : JNCCN*.

[11] Riemsma R. P., Bala M. M., Wolff R., Kleijnen J., Cochrane Hepato-Biliary Group (2013). Transarterial (chemo)embolisation versus no intervention or placebo intervention for liver metastases. *The Cochrane Database of Systematic Reviews*.

[12] Cosimelli M., Golfieri R., Cagol P. P. (2010). Multi-centre phase II clinical trial of yttrium-90 resin microspheres alone in unresectable, chemotherapy refractory colorectal liver metastases. *British Journal of Cancer*.

[13] Hughes E. S. R., Cuthbertson A. M. (1962). Recurrence after curative excision of carcinoma of the large bowel. *JAMA*.

[14] Vatandoust S., Price T. J., Karapetis C. S. (2015). Colorectal cancer: metastases to a single organ. *World Journal of Gastroenterology*.

[15] Mitry E., Guiu B., Cosconea S., Jooste V., Faivre J., Bouvier A. M. (2010). Epidemiology, management and prognosis of colorectal cancer with lung metastases: a 30-year population-based study. *Gut*.

[16] Weiss L., Grundmann E., Torhorst J. (1986). Haematogenous metastatic patterns in colonic carcinoma: an analysis of 1541 necropsies. *The Journal of Pathology*.

[17] Robinson J. R., Newcomb P. A., Hardikar S., Cohen S. A., Phipps A. I. (2017). Stage IV colorectal cancer primary site and patterns of distant metastasis. *Cancer Epidemiology*.

[18] Pei J. P., Zhang C. D., Fan Y. C., Dai D. Q. (2019). Comparison of different lymph node staging systems in patients with resectable colorectal cancer. *Frontiers in Oncology*.

[19] National Comprehensive Cancer NetworkClinical Practice Guidelines in Oncology (NCCN Guidelines®), Colon Cancer.

[20] Mayo E., Llanos A. A. M., Yi X., Duan S. Z., Zhang L. (2016). Prognostic value of tumour deposit and perineural invasion status in colorectal cancer patients: a SEER-based population study. *SEER-Based Population Study*.

[21] Ueno H., Mochizuki H. (1997). Clinical significance of extrabowel skipped cancer infiltration in rectal cancer. *Surgery Today*.

[22] Puppa G., Maisonneuve P., Sonzogni A. (2007). Pathological assessment of pericolonic tumor deposits in advanced colonic carcinoma: relevance to prognosis and tumor staging. *Modern Pathology*.

[23] Wong-Chong N., Motl J., Hwang G. (2018). Impact of tumor deposits on oncologic outcomes in stage III colon cancer. *Diseases of the Colon and Rectum*.

[24] Li Y., Zhao L., Güngör C. (2019). The main contributor to the upswing of survival in locally advanced colorectal cancer: an analysis of the SEER database. *Therapeutic Advances in Gastroenterology*.

[25] Baltatzis M., Chan A. K. C., Jegatheeswaran S., Mason J. M., Siriwardena A. K. (2016). Colorectal cancer with synchronous hepatic metastases: systematic review of reports comparing synchronous surgery with sequential bowel-first or liver-first approaches. *European Journal of Surgical Oncology*.

[26] Lykoudis P. M., O'Reilly D., Nastos K., Fusai G. (2014). Systematic review of surgical management of synchronous colorectal liver metastases. *The British Journal of Surgery*.

[27] Li Y., Liu W., Pei Q. (2019). Predicting pathological complete response by comparing MRI-based radiomics pre- and postneoadjuvant radiotherapy for locally advanced rectal cancer. *Cancer Medicine*.

[28] Liu W., Li Y., Zhu H. (2019). The relationship between primary gross tumor volume and tumor response of locally advanced rectal cancer: pGTV as a more accurate tumor size indicator. *Journal of Investigative Surgery*.

[29] Brulé S. Y., Jonker D. J., Karapetis C. S. (2015). Location of colon cancer (right-sided versus left-sided) as a prognostic factor and a predictor of benefit from cetuximab in NCIC CO.17. *European Journal of Cancer*.

[30] Moretto R., Cremolini C., Rossini D. (2016). Location of primary tumor and benefit from anti-epidermal growth factor receptor monoclonal antibodies in patients with RAS and BRAF wild-type metastatic colorectal cancer. *The Oncologist*.

[31] Loupakis F., Yang D., Yau L. (2015). Primary tumor location as a prognostic factor in metastatic colorectal cancer. *Journal of the National Cancer Institute*.

[32] Nordholm-Carstensen A., Krarup P. M., Jorgensen L. N., Wille-Jørgensen P. A., Harling H., Danish Colorectal Cancer Group (2014). Occurrence and survival of synchronous pulmonary metastases in colorectal cancer: a nationwide cohort study. *European Journal of Cancer*.

[33] Oweira H., Mehrabi A., Reissfelder C., Abdel-Rahman O. (2020). A real-world, population-based analysis of the outcomes of colorectal cancer patients with isolated synchronous liver or lung metastases treated with metastasectomy. *World Journal of Surgery*.

